# Antibacterial fatty acids: An update of possible mechanisms of action and implications in the development of the next-generation of antibacterial agents

**DOI:** 10.1016/j.plipres.2021.101093

**Published:** 2021-02-09

**Authors:** Giancarlo Casillas-Vargas, Carlimar Ocasio-Malavé, Solymar Medina, Christian Morales-Guzmán, René García Del Valle, Néstor M. Carballeira, David J. Sanabria-Ríos

**Affiliations:** a Faculty of Science and Technology, Department of Natural Sciences, Inter American University of Puerto Rico, Metropolitan Campus, PO Box 191293, San Juan, PR 00919, USA; b University of Puerto Rico, Río Piedras Campus, Department of Chemistry, 17 Ave. Universidad Ste. 1701, San Juan, PR 00925-2537, USA

**Keywords:** Antibacterial agents, Fatty acids, Non-traditional antibacterial, FA mechanisms, Multidrug-resistant bacteria

## Abstract

The antibacterial activity of fatty acids (FA) is well known in the literature and represents a promising option for developing the next-generation of antibacterial agents to treat a broad spectrum of bacterial infections. FA are highly involved in living organisms’ defense system against numerous pathogens, including multidrug-resistant bacteria. When combined with other antibacterial agents, the remarkable ability of FA to enhance their bactericidal properties is a critical feature that is not commonly observed in other naturally-occurring compounds. More reviews focusing on FA antibacterial activity, traditional and non-traditional mechanisms and biomedical applications are needed. This review is intended to update the reader on the antibacterial properties of recent FA and how their chemical structures influence their antibacterial activity. This review also aims to better understand both traditional and non-traditional mechanisms involved in these recently explored FA antibacterial activities.

## 1. Introduction

Fatty acids (FA) are organic compounds with carboxylic acids, with long aliphatic chains that may be straight or branched, saturated, or unsaturated [1]. The antimicrobial properties of FA have been known for a long time, and, interestingly, FA are produced by plants and algae to defend themselves against pathogens, including multidrug-resistant bacteria (MDRB) [2–4]. The fact that FA display antibacterial activity against MDRB could be relevant for the Centers for Disease Control and Prevention (CDC) since these compounds could represent the next-generation of antibacterial agents to treat and prevent bacterial infections [5–7]. Although several reviews describe the antibacterial activity of FA [3,4,7,8], the apparent multiple mechanisms that confer antibacterial activity to FA is not well understood and needs to be further reviewed. Moreover, FA have been tested in combination with other antibiotics, becoming practical their use [4]. A recent example of FA’s synergistic effect combined with penicillins, fluoroquinolones, and aminoglycosides in Gram-positive and Gram-negative bacteria was reported in the literature [9,10]. Also, FA have shown remarkably anti-inflammatory and wound healing effects [11–13]. Taken together, these results make FA attractive entities for developing novel antibacterial agents.

Understanding the chemical structure of FA is essential for comprehending their antibacterial activities and the multiple mechanisms that can help to develop novel antibacterial compounds. It is imperative to point out that most of the experimental observations with FAs described in this review were carried out *in vitro*. This review aims to identify those chemical characteristics of FA responsible for the antibacterial activity towards Gram-positive and Gram-negative bacteria and identify possible targets in the mechanisms responsible for the antibacterial activities of the most unusual FA.

## 2. Antibacterial Properties of Saturated Fatty Acids (SFA)

**Fig. 1.**
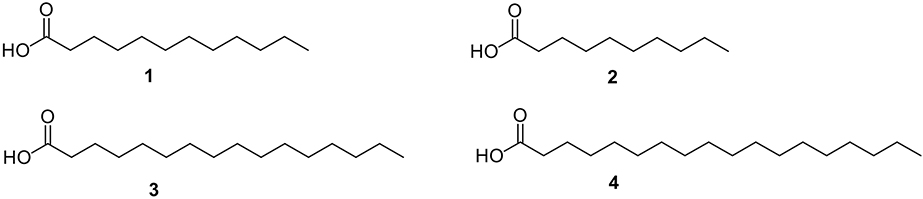
Chemical structures of lauric acid (**1**), capric acid (**2**), palmitic acid (**3)**, and stearic acid (**4**).

It is well known that several SFA have shown remarkable antibacterial properties towards Gram-positive and Gram-negative bacteria (Fig. 1) [12,14–16], which could represent an alternative for the development of novel antibacterial agents. Among the diversity of saturated FA, lauric acid (**1**) is a naturally-occurring FA with significant antibacterial activity. Acid **1** has been tested against a wide range of bacteria, having a higher effect on Gram-positive bacteria [17,18]. A 1:10 ν/v dilution of **1** exhibited growth inhibition against clinical isolates of *Staphylococcus aureus* and *Streptococcus pneumoniae*, displaying inhibition zones of 15 mm, while towards *Mycobacterium tuberculosis, Escherichia coli,* and *Salmonella* spp., acid **1** displayed the lowest activity obtaining inhibition zones of 8 mm [17]. Another study demonstrated that **1** was antibacterial against *Streptococcus agalactiae, Streptococcus mutans, Streptococcus pyogenes, Streptococcus salivarius, Streptococcus sanguinis,* and *S. aureus* showing inhibition zones between 14 and 58 mm at a concentration of 0.085 μmol [18]. Additionally, this study demonstrated that **1** was not active against Gram-negative *E. coli*, *Klebsiella oxytoca*, *Klebsiella pneumoniae*, *Pseudomonas aeruginosa,* and *Serratia marcescens*. Nitbani and colleagues reported that 15% ν/v of **1** showed higher antibacterial activity towards *S. aureus*, *Bacillus cereus, Salmonella typhimurium,* and *E. coli* than 0.5% ν/v of Ciprofloxacin [16]. In a study performed by Yoon and colleagues, **1** exhibited a membrane disruptive behavior and inhibited the growth of susceptible strains of *S. aureus* and methicillin-resistant *S. aureus* (MRSA) [19]. In addition, **1** has shown potent antimicrobial activity against *Clostridium difficile* isolates [12]. Lauric acid (**1**) inhibits the growth of the bacteria mentioned above by membrane disruption and induces reactive oxygen species production, magnifying the outgrowth inhibition effect on sodium taurocholate spores [12]. In the same study, a mouse infection model was used, and a reduction of *C. difficile* infection symptoms was observed and a decrease in proinflammatory cytokine production [12].

The potential of capric acid (**2**) as an antibacterial agent has also been explored and compared with **1**. In terms of chemical structure, **2** is just two carbons shorter than **1**. However, **1** still shows more potency as an antibacterial agent than **2** towards *Propionibacterium acnes*, both *in vitro* and *in vivo* [14]. The apparent antibacterial effect of **2,** and other medium-chain FA (MCFA, FA that contain 6–12C) present in essential oils, has been reported towards the highly pathogenic bacterium *E. coli* O157: H7 [20]. In that study, the reduction of viable bacterial cells was observed when *E. coli* was treated with either extract from the essential oils or with **2** alone. It was particularly interesting to observe that the essential oils’ antibacterial activity was higher than either **1** or **2** alone, suggesting that these SFA could be exerting a synergetic effect with other chemical components present in the essential oil extracts.

Palmitic acid (**3**) and stearic acid (**4**) are two other SFA examples that display antibacterial activity towards Gram-positive and Gram-negative bacteria. Acid **3** has a 16-carbon chain length in its chemical structure, while **4** has an 18-carbon chain length. Interestingly, nanostructure arrays of **3** and **4** have been obtained from recrystallization of the surface of highly ordered pyrolytic graphite and successfully tested against *P. aeruginosa* and *S. aureus* [15]. These SFA were encapsulated in liposome carriers showing bactericidal activity against multidrug-resistant *Staphylococcus epidermidis* and Vancomycin-resistant *Enterococcus faecalis* [21]. In the case of liposomal **3**, this drug delivery system displayed MIC values of 0.5 μg/mL towards *S. epidermis* and 2 μg/mL towards *E. faecalis*, while liposomal **4** displayed MIC values of 0.25 μg/mL towards *S. epidermis* and 0.5 μg/mL towards *E. faecalis* [21].

Other research groups have reported that **3** is not antibacterial against Gram-positive or Gram-negative bacteria [9,22,23]. These differences may be due to the type of bacteria used in these studies. Not all bacteria show the same behavior when exposed to a particular treatment. Another reason for this discrepancy in the literature could be the use of liposomes as drug carriers, which could facilitate the delivery of **3**.

## 3. Antibacterial properties of unsaturated fatty acids (uFA)

Unsaturated fatty acids (uFA) are medium- or long-chain carboxylic acids that contain one or more double bonds. These FA can have double bonds with either the *cis* or *trans* stereochemistry. Although not common, there are uFA that contain ring moieties in their aliphatic chains. The literature has reported that some uFA display inhibitory activity against Gram-positive and Gram-negative bacteria [3,4,7,8]. This review will cover some of these uFA that possess biological activity against bacteria and their potential use as the next-generation of antibacterial agents. Additionally, we will discuss the ability of uFA to afford a synergetic effect with current antibiotics.

### 3.1. Antibacterial uFAs containing double bonds

**Fig. 2.**
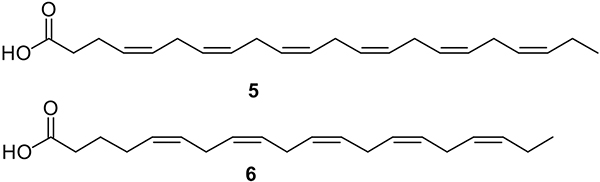
Chemical structures of DHA (**5**) and EPA (**6**).

uFA are medium- and long-chain carboxylic acids that contain double bonds (C=C) in their chemical structures. These uFA are divided into monounsaturated FA (MUFA, with one double bond) and poly-unsaturated fatty acids (PUFA, with two or more double bonds). uFA have displayed noteworthy antibacterial activities in many pathogens, including *Pseudomonas aeruginosa, Porphyromonas gingivalis, Fusobacterium nucleatum, Neisseria gonorrhoeae*, and *Helicobacter pylori* [10,24–26]. In 2016, Sun and colleagues isolated docosahexaenoic acid (DHA, **5**) and eicosapentaenoic acid (EPA, **6**) from fish oil and demonstrated that these PUFA (Fig. 2) displayed inhibitory activity against *P. gingivalis* and *F. nucleatum* [26]. Both DHA and EPA showed inhibition of *P. gingivalis* at a MIC of 12.5 μM, while for *F. nucleatum,* these FA displayed MIC values greater than 100 μM. Also, DHA and EPA inhibited biofilm formation at a MIC of 12.5 μM in *P. gingivalis*, while in *F. nucleatum,* only EPA was able to inhibit the biofilm formation at a MIC of 100 μM (Sun et al., 2016). Moreover, it was demonstrated that DHA and EPA were not cytotoxic towards the tissue cells hGFs and hPDLCs [26]. In a similar study, Sun et al. (2017) investigated the bactericidal activity of DHA and EPA against *Streptococcus mutans* [27]. Both PUFA showed a bacteriostatic effect at MIC values of 100 μM and 50 μM, respectively, and reduced the thickness of a biofilm at 100 μM. Additionally, in 100 μM, the EPA downregulated the expression of the genes *gtfB, ftf, gbpB, vicR, brpA, smu630,* and *comDE*, which are associated with biofilm formation. On the other hand, DHA showed less activity, downregulating only *gtfB, ftf, vicR, smu630,* and *comDE* [27]. Moreover, the antibacterial activity of EPA was successfully tested against the pathogens *B. cereus* and *S. aureus* showing a minimum inhibitory concentration (MIC) value of 64 mg/L for both bacteria [28]. Nevertheless, the minimum bactericidal concentration (MBC) for *B. cereus* was 64 μg/mL, while for *S. aureus* MBC the value was 128 μg/mL, showing more potency against *Bacillus cereus*. The referred study demonstrated that inhibitory concentrations of EPA did not lead to the emergence or selection of strains with reduced susceptibility [28].

**Fig. 3.**
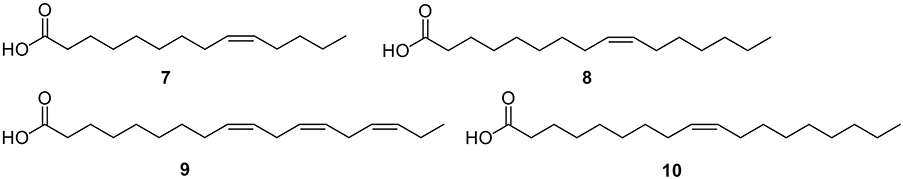
Chemical structures of myristoleic acid (**7**), palmitoleic acid (**8**), α-linolenic acid (**9**), and oleic acid (**10**).

uFA have also shown bioactivity against *Opthalmia neonatorum*, a term used to refer to conjunctivitis occurring in newborns afflicted by pathogens, especially *N. gonorrhoeae*. Churchward and colleagues screened 37 FA and derivatives to develop FA-based prophylaxis to treat *N. gonorrhoeae* eye infections [24]. They found that myristoleic acid (**7**, Fig. 3) not only inhibited the pathogen growth, but it was a fast-acting and nonirritating agent. As a result, **7** was the best compound tested to treat infections localized in the eyes [24]. In another study, FA-based microemulsions were prepared to treat *S. aureus,* a common pathogen that causes *Opthalmia neonatorum* conditions. Among the FA assayed, the most effective against *S. aureus* were **7**, palmitoleic acid (**8**), and α-linolenic acid (**9**, Fig. 3). However, only **9** was selected for the microemulsions because of its remarkable activity and cost-effectiveness, demonstrating its potential as a novel treatment against *Opthalmia neonatorum* [29]. Nevertheless, further aspects of using different carriers to enhance FA’s effect in pathogenic bacteria resistant strains is a path yet to be explored.

New resistant strains have been added to the list of commonly occurring bacteria, like *H. pylori* [25]. However, the novel synthesis of liposomal linolenic acid (**9**) was effective against *H. pylori* with MBC value of 200 μg/mL [30]. In contrast, liposomal **4** showed a higher MBC at 1 mg/mL, and liposomal oleic acid (**10**, this FA structure is displayed in Fig. 3) did not display an antibacterial effect on *H. pylori*. Furthermore, in a recent study by Selvadoss and collaborators, liposomal **10** containing antibiotics were prepared and tested on 32 strains of multidrug-resistant *P. aeruginosa* [10]. The liposomal **10** was loaded with the following antibiotics: cefepime (CPM), ciprofloxacin (CIP), ceftazidime (CAZ), ampicillin (AMP), piperacillin (PIP), cephalexin (CN), amikacin (AK), imipenem (IPM), tobramycin (TOB), gentamycin (GEN), ceftriaxone (CTX), and nitrofurantoin (NIT). When the *P. aeruginosa* strains were treated with the liposomal formulation of the antibiotics mentioned above, MIC values reached up to 4-fold lower than the free antibiotics. Therefore, liposomal **10** restored the susceptibility of these multidrug-resistant *P. aeruginosa* strains to the antibiotics mentioned above [10].

The successful bactericidal activity of DHA loaded into a nanostructured lipid carrier (NCL) has been reported [31]. Seabra and colleagues nano-encapsulated DHA to improve its bactericidal activity towards *H. pylori*, thus avoiding the loss of activity of DHA by oxidation and making DHA more efficient as an antibacterial agent. The results demonstrated that **5**-loaded NCL was able to inhibit *H. pylori* growth at a MIC of 10 μM, while free DHA only showed a MIC of 100 μM. They also reported that DHA-loaded NCL was cytotoxic towards the MKN45 cell line at 100 μM [31].

### 3.2. Antibacterial properties of FA containing rings

Marine microorganisms are a never ending rich source of biologically active compounds. They have recently been described as a “particularly promising” source for searching new antimicrobials to combat antibiotic-resistant strains [32]. Therefore, secondary marine bacterial metabolites have become an excellent source of novel antibiotics [33,34]. *Labrenzia* sp. 011 is a marine bacterium that can produce two cyclopropanes containing FA, cis-4-(2-hexylcyclopropyl)-butanoic acid (**11**), and cis-2-(2-hexylcyclopropyl)-acetic acid (**12**). When testing disks were impregnated with 50 μg of **11**, inhibition zones of 2 mm in *E. coli* and 5 mm in *Pseudoroseovarius crassostreae* (the causative agent of Roseovariusoyster disease) were observed. In the same study, **12** showed inhibition zones of 3 mm in *E. coli*, 10 mm in *Bacillus megaterium*, and 5 mm in *P. crassostreae* when disks were impregnated with 50 μg of the acid. Compound **12** was particularly toxic against the multidrug-resistant *E. coli* I-11276b displaying an inhibition zone of 2 mm, MRSA LT-1338 displaying an inhibition zone of 2 mm, and MRSA LT-1334 showing an inhibition zone of 3 mm when disks were impregnated with 4 μg of the FA [34].

The novel furan fatty acid, 7,10-epoxyoctadeca-7,9-dienoic acid (7,10-EODA, **13**), inhibits several Gram-positive bacteria, among them, six MRSA strains at MIC values of 125–250 μg/mL [35]. However, this uFA did not inhibit *P. aeruginosa*, *E. coli*, and *S. thyphimurium*. Acid **13** also showed enzymatic inhibitory activity against MRSA virulence factors such as hemolysin, protease, and autolysin enzymes [35]. In a more recent study, Dasagrandhi et al. reported the use of **13** as an adjuvant for β-lactam antibiotics against multidrug-resistant *S. aureus* (MDRSA) [36]. The combination of both compounds exhibited a better range of inhibition than **13** alone. This outcome is of great interest since bacterial resistance to a particular antibiotic can be avoided when combined with **13**. Table 1 provides relevant information regarding uFA, including their chemical structures, source, type of formulation, target bacteria, and inhibitory activity range.

### 3.3. Acetylenic FA (aFA) and their application as antibacterial agents

Acetylenic fatty acids (aFA) are medium- or long-chain carboxylic acids that contain one or more triple bonds. The literature has described that aFA display interesting antibacterial activity towards nosocomial pathogens [9,22,23,37]. Moreover, it was reported that the triple bond position in the carbon chain plays a critical role in the antibacterial activity of the aFA. For example, Sanabria-Ríos and colleagues demonstrated that the triple bond at C-2 in a C16-aFA is pivotal for its antibacterial activity [37]. More recently, this group demonstrated that the bactericidal activity of aFA against MRSA decreases as the triple bond is moved farther from the carboxyl group [9]. The aFA that was extensively studied by Sanabria-Ríos’ research group is the 2-hexadecynoic acid (**14**). The antibacterial properties of **14** were tested against a wide range of microorganisms, including *S. aureus*, *Staphylococcus saprophyticus*, *B. cereus*, *Klebsiella pneumoniae,* and *P. aeruginosa* [37]. In a recent study, Sanabria-Ríos et al. assessed the bactericidal properties of **14** against CIMRSA strains resistant to CIP, demonstrating its effectiveness in inhibiting bacterial growth. They proved that the antibacterial activity of **14** was higher than CIP. Sanabria-Ríos’ research team also demonstrated that the combination of **14** with CIP improved the antibacterial activity of CIP in ciprofloxacin-resistant *S. aureus* (CRSA) strains [9].

In another study performed in 2015, Sanabria-Ríos and colleagues synthesized C5 curcumin-**14** conjugates to determine whether the chemical conjugation of **14** to C5-curcumin (C5-Curc) enhances the antibacterial activity of C5-Curc in CIMRSA [23]. This author demonstrated that the presence of **14** enhances the antibacterial activity of C5-Curc against eight CIMRSA strains obtaining IC_50_ values ranging between 24.9 and 39.5 μg/mL. Despite the enhancing antibacterial effect of the C5-Curc-**14** conjugate, **14** is still more effective alone than the conjugated C5-Curc-**14**. Indeed, **14** displays potent antibacterial activity, which can be further investigated as an antibacterial agent that can be used alone or in combination with other more classical antibiotics.

Bactericidal properties of naturally occurring aFA have also been recently reported. Liu and colleagues were able to isolate aFA from ethanolic extracts of the herb *Thesium chinese*, an Asian plant commonly used for oral treatments [38]. Among all the investigated extracts, they found exocarpic acid (**15**, Table 2) to have a more significant antibacterial effect against *P. gingivalis* (MIC = 0.86 μg/mL), *F. nucleatum* (MIC = 3.40 μg/mL), and *S. mutans* (MIC = 13.70 μg/mL).

The α-methoxylated FA are another type of uFA that have shown significant antibacterial activity, and many of these FA were isolated from Caribbean sea sponges [22,39]. In 2002, Carballeira and colleagues identified in Caribbean sponges α-methoxylated FA between 14 and 28 carbons, with double or triple bonds, and with *iso-anteiso* methyl branches in their chemical structures [39]. They also reported that synthetic α-methoxylated FA displayed inhibitory activity towards Gram-positive and Gram-negative bacteria [22]. That was the case of (±)-2-methoxy-6-hexadecynoic acid (**17**) and (±)-2-methoxy-6-octadecynoic acid (**18**), which displayed significant activity against *S. aureus*, CIMRSA, and *E. coli* at IC_50_ values ranging between 30 and 500 μg/mL [22]. Ironically, it was also demonstrated that the presence of a double bond at C-6 in the FA favored the antibacterial activity of the α-methoxylated FA. Therefore, these findings suggested that the C-6 triple bond in the chemical structure of the α-methoxylated FA was pivotal for their antibacterial activity.

More recently, Carballeira and collaborators performed a structure-activity relationship (SAR) study with a complete series of α-methoxylated and non-methoxylated C14 FA (unpublished results). The α-methoxylated 2-methoxy-6-tetradecynoic acid (**19**), the naturally-occurring 2-methoxy-6*Z*-tetradecenoic acid (**20**) as well as the also naturally occurring 2-methoxytetradecanoic acid (**21**) were synthesized from simple precursors to determine their antibacterial activity towards six clinical isolates of methicillin-resistant *Staphylococcus aureus* (CIMRSA). The 6-tetradecynoic acid (**22**) and 6*Z*-tetradecenoic acid (**23**) were also included in this study for a better structure-activity comparison. The best antimicrobial acid in the series was **19**, which displayed an IC_50_ between 30 and 48 μg/mL, followed by **23** with IC_50’s_ between 34 and 130 μg/mL (unpublished results). Both α-methoxylated acids **20** and **21** were not effective at all with IC_50’s_ between 105 and 380 μg/mL as well as the non-methoxylated acetylenic acid **22** with IC_50’s_ between 104 and 286 μg/mL. These results clearly indicate that both the presence of the α-methoxylated functionality and the triple bond at C-6 were instrumental for the leading antimicrobial activity displayed by **19**. It is important to mention that all these FA were not antimicrobial towards *E. coli* (IC_50_’s > 1,000 μg/mL). Recent studies from our laboratories also determined that **19** displays the lowest critical micelle concentration (CMC) of the series at 70–90 μg/mL, followed by **23** with a CMC > 100 μg/mL. These results tend to indicate that the antibacterial activity of the acetylenic methoxylated FA might be mediated by micellar aggregation. Table 2 provides a good summary of the aFA that have displayed antibacterial activity against Gram-positive and Gram-negative bacteria.

## 4. Possible antibacterial mechanisms of action of these novel FA

### 4.1. Traditional mechanisms that are the target for antibacterial FA

Traditional mechanisms for common antibiotics are classified as follows: inhibition of DNA/RNA replication (ciprofloxacin, norfloxacin, novobiocin, and rifampin) [40], cell wall synthesis (amoxicillin, cefalexin, and oxacillin among many) [41], protein synthesis (chloramphenicol, clarithromycin, and erythromycin) [42], disruption of the cytoplasmic membrane (polymyxin B, daptomycin) [43], and inhibition of metabolic routes (sulfonamides, sulfones, trimethoprim, and isoniazid) [44]. This part of the review will explain several FA antibacterial activities in terms of the known mechanisms of action of traditional antibacterial agents.

#### 4.1.1. DNA/RNA replication inhibitors

DNA replication is essential for cell viability, representing an attractive target for the development of novel antimicrobials [40]. For that reason, the search for novel compounds with the ability to inhibit this process is critically needed. uFA can inhibit bacterial growth through the inhibition of DNA/RNA replication [9,23]. For example, **14** displayed inhibitory activity against DNA gyrase [9], an essential enzyme that controls the topological state of DNA replication [45]. Moreover, it was also demonstrated that the chemical conjugation of **14** to C5-Curc improved the effect of C5-Curc in inhibiting the supercoiling activity of DNA gyrase [23].

#### 4.1.2. Cell wall biosynthesis inhibitors in Gram-positive bacteria

**Fig. 4.**
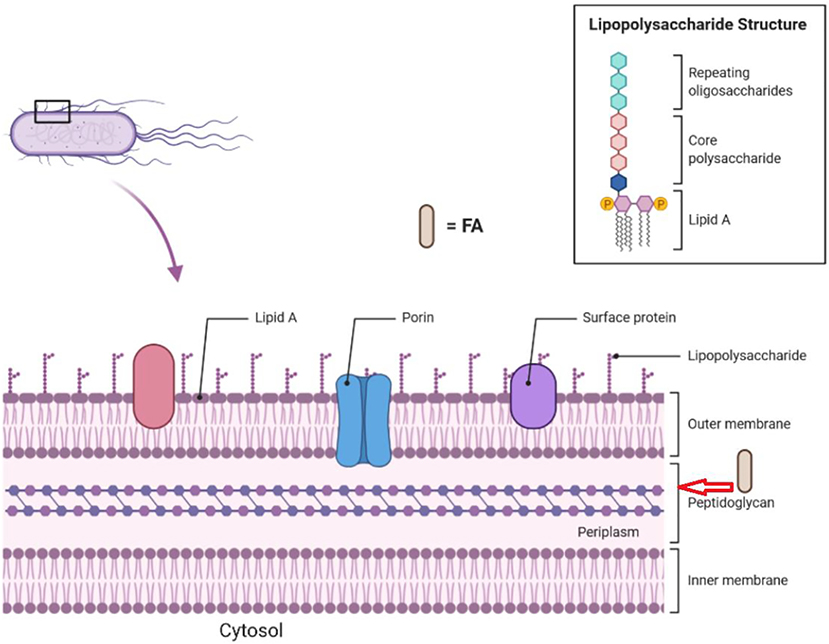
General representation of the components of a Gram-negative bacterial cell wall that could be affected by FAs. Images were created with BioRender.com

**Fig. 5.**
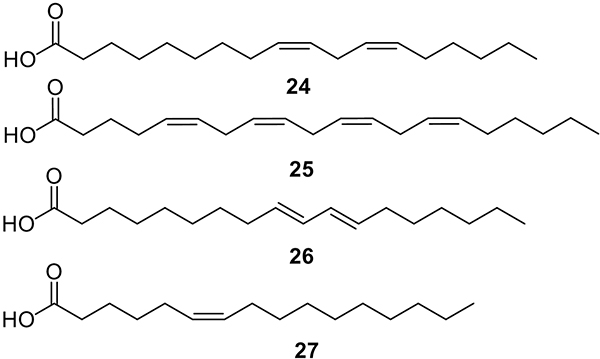
Structures of linoleic acid (**24**), arachidonic acid (**25)**, conjugated linoleic acid (**26**), and *cis*-6-hexadecenoic acid (**27**).

The bacterial cell wall is a vital structure to maintain osmotic pressure, cell shape, and cell integrity, which are pivotal for bacterial viability [46]. Peptidoglycan (PG) is an essential molecule that forms part of the cell wall found outside the cytoplasmic membrane of almost all bacteria (Fig. 4) [47]. PG is a heteropolymer composed of glycan strands that are crosslinked with peptides. The glycan backbone is composed of alternating *N*-acetylglucosamine units and *N*-acetylmuramic acids linked by β−1,4-glycosidic bonds [48]. In 2009, Kenny and colleagues determined that linoleic acid (**24**, Fig. 5) can alter PG synthesis genes in *S. aureus* [49]. Upregulation of PG precursors such as pentaglycine, lysine, glutamate, D- alanine, L- alanine, and teichoic acid in the presence of **24** could represent a response of the cell to PG inhibition [49]. In another study performed by Zheng and colleagues, it was reported that **24** displayed potent and selective inhibition of FabI, an essential protein in the FA biosynthesis in *S. aureus* and *E. coli* [50]. This study also reported that **8**, **24**, and arachidonic acid (**25**, Fig. 5) showed FabI inhibition, whereas their corresponding SFA were not active against the enzyme mentioned above.

#### 4.1.3. Inhibitors of protein synthesis

Proteins are the cells working machinery that carries out most cellular work and provides many essential bacterial subsistence functions. Thus, inhibiting their biosynthesis may represent an attractive target for developing novel antibacterial agents [42]. Compound **24** has shown exceptional activity against *Vibrio cholerae*. This uFA has inhibitory activity on the binding ability of ToxT, a DNA binding protein that activates the transcription of major virulence genes that encode cholera toxin and toxin-coregulated pilus [51]. Acid **24** shows a more significant equilibrium dissociation constant (K_D_), representing the concentration of ToxT required for binding 50% of the DNA at equilibrium, at the ToxT promoters *tagA* and *tcpP* with K_D_ values of 27.78 and 171.7, respectively. The activity of **24** at the promoters *tcpA* (which contains two ToxT binding sites) and aldA (which contains one binding site) showed K_D_ values of 452.6 and 48.67, respectively. While Virstatin, a synthetic ToxT inhibitor, which affects promoters with only two binding sites, showed K_D_ values of 27.24 and 11.79, respectively [51]. These results suggested that **24** is a better ToxT binding inhibitor than Virstatin. Similar results were obtained with a conjugated form of **24** (conjugated linoleic acid **26**, Fig. 5), which showed a greater K_D_ for the ToxT promoter tcpA than the bacteria without treatment, resulting in inhibition of the cholera toxin production [52]. In the same study, *in vivo* effects of **24** against *V. cholerae* were assessed using the rabbit ileal loop model, an assay that consists of 10-cm loops of small intestine getting injected with the treatment and/or the pathogen. Cholera toxin levels were significantly reduced (< 200 ng/mL) when 400 μL of **26**, diluted with 10% Kollidon (a polyvinylpyrrolidone polymer) in a total volume of 1 mL was administered, while the control showed > 1200 ng/mL. The fluid accumulation decreased <0.2 mL/cm compared to control, > 1.4 mL/cm, suggesting it could reduce secretory diarrhea [52].

#### 4.1.4. Cytoplasmic membrane disruption

At the frontier of life, the cell membrane is one of the cell’s most essential structures. The cytoplasmatic membrane’s prominence relies on the many essential functions, making it one of the main targets when developing new antibacterial therapies. In a recent study, Sun and collaborators reported that the PUFA **5** and **6** provoked membrane disruption in bacteria. With the use of scanning electron microscopy (SEM), they observed that these two PUFA could completely disrupt the cell membrane of *P. gingivalis* [26]. Additionally, morphological changes, including much rougher bacterial membranes, have been observed in *S. mutans* when treated with either **5** or **6** [27]. Moreover, Le and Debois reported that **6** showed disruptive membrane activity against *B. cereus* and *S. aureus* [28]. They quantified the leakage of 260-nm absorbing material, including genetic material, from the bacterial cells in suspension and showed an increase of absorbing material in the extracellular space as the concentration of **6** increased, suggesting membrane disruption.

Another FA displaying disruptive membrane properties is lauric acid (**1**). Yang et al. investigated the effect of **1** on the integrity of the cytoplasmatic membrane of *C. difficile* [12]. In this study, it was discovered that *C. difficile* treated with **1** released a higher concentration of nucleic acid than an untreated control, which suggests that **1** compromised the bacterial membrane integrity. These results were confirmed by ultrathin-section transmission electron microscopy (TEM) when abnormal cell morphology and cytoplasmic content leakage was observed. Similarly, **1** showed abnormal cell morphology on *S. aureus* and MRSA by inducing a tubular formation on the lipid bilayer, ultimately resulting in cell lysis [19].

Other MDRSA strains have also been inhibited by β-lactam antibiotics when the furan acid **13** was used as an adjuvant [36]. This FA alters membrane integrity allowing and facilitating antibiotic uptake. The MIC value of oxacillin and ampicillin were reduced by 4- to 16-fold when combined with **13**. Additionally, the MIC of penicillin was reduced by 2- to 16-fold in the presence of **13**.

The *cis*-6-hexadecenoic acid (**27**, Fig. 5) is another FA displaying an inhibitory effect on *S. aureus* [53]. Acid **27** has shown a substantial ability to kill *S. aureus*, even at low concentrations. In terms of the antibacterial mechanism, it was reported that **27** causes loss of membrane integrity through the disruption of the proton motive force, an increase in membrane fluidity, and the electron transfer pathways [53].

**Fig. 6.**
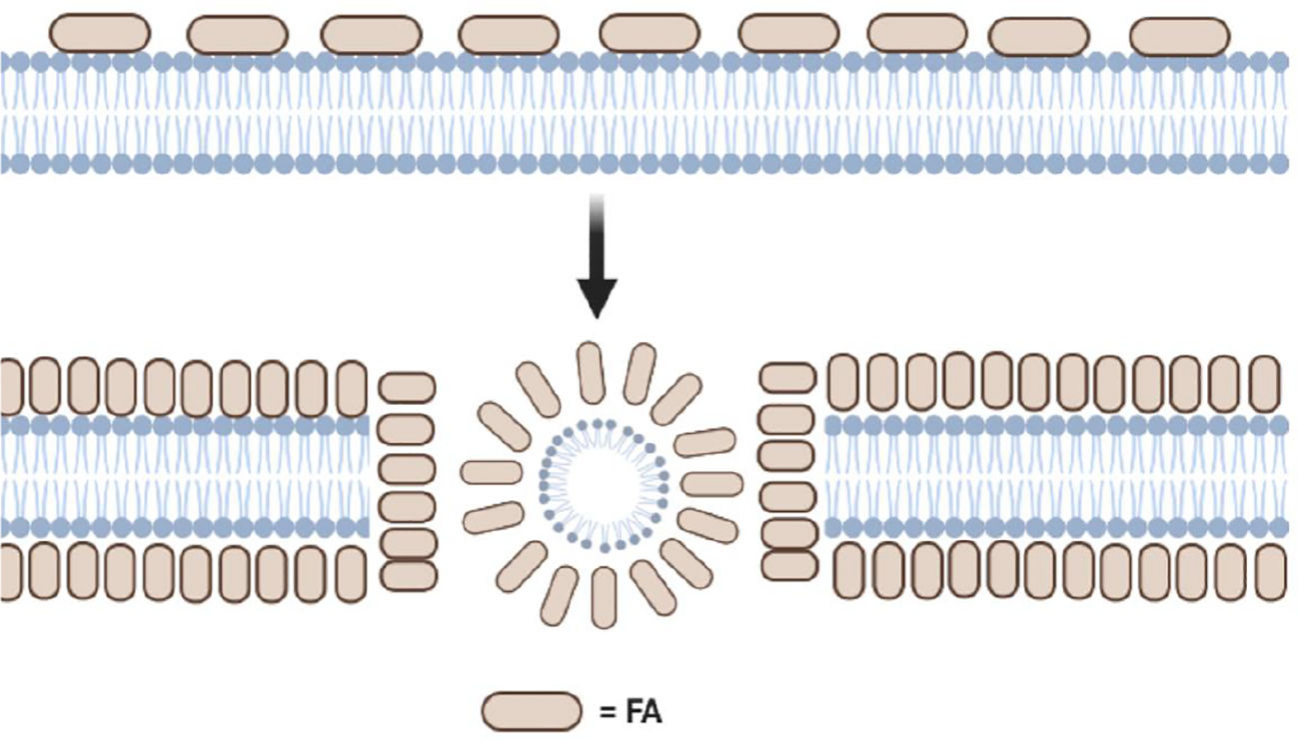
A BioRender-generated image showing the conceptualization of how FA could induce cell membrane disruption. FA, which are represented by small ovals (in ivory), could interact with the phospholipids of the cell membrane (in light blue) forming lipid micelle aggregates. These micelles could encapsulate part of the cell membrane’s phospholipid content thus compromising the bacterial cell membrane integrity. (For interpretation of the references to colour in this figure legend, the reader is referred to the web version of this article.)

Other studies involving liposomal **9** and liposomal **10** have shown that these uFA formulations promote membrane permeability on clinical isolated antibiotic-resistant strains of *H. pylori* [30]. However, the cells treated with liposomal **9** showed more ATP release than liposomal **10**, indicating that liposomal **9** induced higher permeability. Morphologically speaking, bacteria treated with liposomal **9** may undergo plasma membrane separation from the outer membrane resulting in loss of cytoplasmic contents, while MDRSA strains treated with liposomal **10** displayed similar morphological changes, although some intact bacteria were also present. Fig. 6 shows a schematic conceptualization of how FA could induce cell membrane disruption.

#### 4.1.5. Inhibitors of metabolic routes

Metabolism comprises energy converting reactions that keep the cell working and alive, making it an ideal target for novel antibiotics development. In *S. aureus*, exogenous FA are taken and converted to an acyl carrier protein, undergo elongation, and are incorporated into the membrane phospholipids [54]. In a study conducted by Parsons and colleagues, it was demonstrated that palmitoleic acid (**8**) is a poor substrate for phospholipid biosynthesis, and therefore it is accumulated in the cell, so it becomes deleterious to the metabolism [55]. The toxic FA triggered disruption of the cell membrane and its functions, *e.g.*, proton gradient, resulting in energy loss. Also, **8** was able to block macromolecular synthesis [55].

Bacterial metabolic routes could also be affected by bacteria exposure to linoleic acid (**24**) [49]. It was reported that **24** causes an alteration in the expression of genes involved in the glycolytic and fermentative metabolic pathways resulting in *S. aureus* loss of energy production.

### 4.2. FA targeting non-traditional mechanisms

**Fig. 7.**
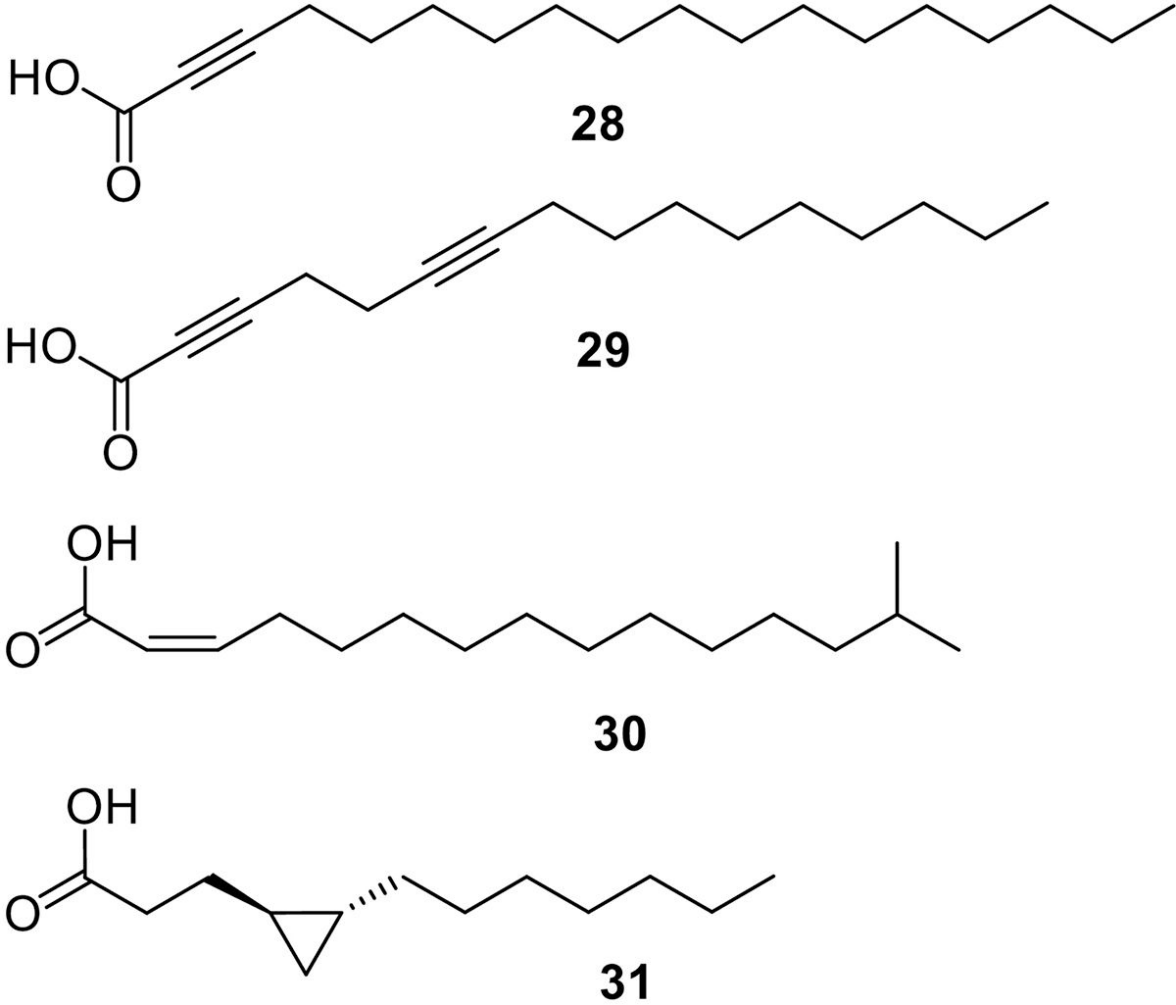
Chemical structures of 2-octadecynoic acid (**28**), 2,6-hexadecadiynoic acid (**29**), cis-14-methyl-2-pentadecenoic acid (**30**), and lyngbyoic acid (**31**).

Non-traditional agents are those compounds that target multiple mechanisms that confer bacterial resistance to antibiotics or provoke the development of virulence instead of directly killing the bacteria. The development of therapeutic agents that target such mechanisms is needed because these agents could reduce antibiotic use and, ultimately, decrease selective pressure on bacteria that favors the evolution of persistence and resistance mechanisms [56]. This section of the review focuses on FA that inhibit non-traditional mechanisms such as horizontal gene transfer (HGT), quorum sensing (QS), and pump efflux of antibiotics. Fig. 7 shows the chemical structures of some FA that display inhibitory effects by targeting the non-traditional mechanisms mentioned above.

#### 4.2.1. Horizontal gene transfer (HGT) inhibition

**Fig. 8.**
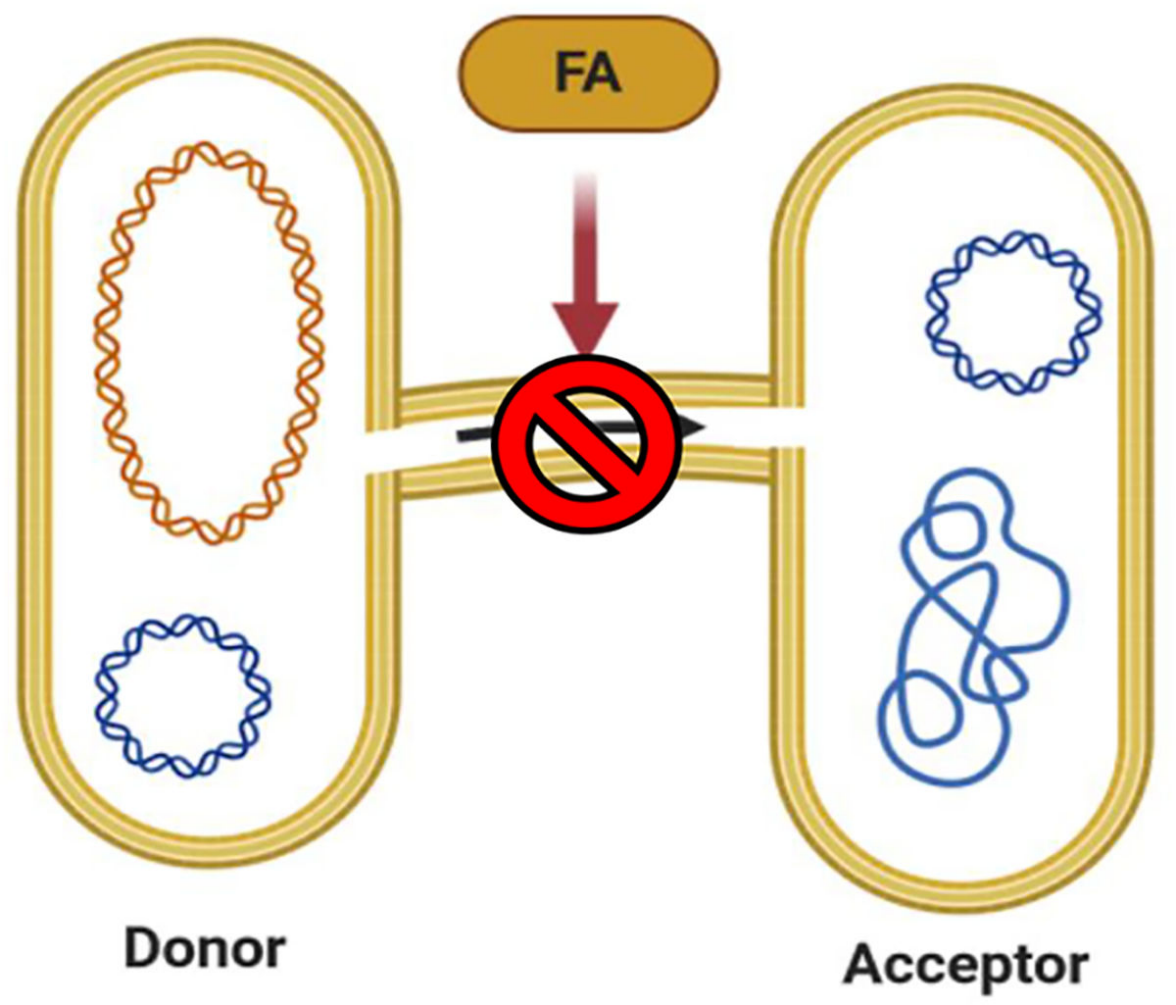
Image showing the conceptualization of bacterial conjugation, a type of HGT process among bacteria. The image was generated with BioRender.com. In this image, a donor bacterium containing an antibiotic resistance plasmid (in blue) transfers this genetic material to an acceptor bacterium that could be susceptible to the antibiotic that the donor bacterium shows resistance. The HGT occurs by forming a protein bridge between the two bacteria known as the conjugation pilus. This image is displaying, in a general way, how FA could disrupt HGT. (For interpretation of the references to colour in this figure legend, the reader is referred to the web version of this article.)

HGT is one of the primary mechanisms used by bacteria to acquire antibiotic resistance [57,58]. This mechanism consists of the “sideways” movement of genetic material through cell-to-cell contact (Fig. 8). This movement is carried out by a conjugative pilus that forms part of the bacterial type IV secretion system (T4SS) [59]. HGT is carried out by related species and unrelated species [60]. Thus, finding molecules that disrupt this process could represent a novel treatment that can be used in combination with the more traditional antibiotics. For example, synthetic aFA such as **14** and 2-octadecynoic acid (**28**, Fig. 7) interfere with HGT [57]. It was found that either **14** or **28** displayed conjugation inhibitory (COIN) activity in *E. coli*, *Salmonella enterica*, *Pseudomonas putida*, and *Agrobacterium tumefaciens*. The main targets of the bacteria mentioned above are the conjugative plasmids IncF, IncW, and IncH [57]. Both **14** and **28** are moderately active against the IncI, IncL/M, and IncX by inhibiting the conjugation and mobilization frequency of these plasmids from the donor cell [57]. Conjugation plasmids are transferred by T4SS, which translocate DNA and proteins to the target cell by direct cell-to-cell contact. It is known that T4SS is a protein complex composed of 11 proteins named sequentially as Virb1, Vir2, Virb3, Virb4, and so on [59]. García-Cazorla et al. demonstrated that **14** targets the traffic ATPase TrwD, a VirB11-homolog in plasmid R388, replacing palmitic acid (**3**) [61]. Failure of TrwD to bind the membrane in *E. coli* results in the inhibition of bacterial conjugation. The FA **10**, **14**, **24**, and 2,6-hexadecadiynoic acid (**29**, Fig. 7) also inhibit the ATPase activity of TrwD in *E. coli* [58].

#### 4.2.2. Quorum sensing (QS) inhibition

QS is a mechanism in which bacteria regulate gene expression in response to population density using signaling molecules [62]. When a certain threshold of extracellular signaling molecule concentration is reached, quorum sensing is activated. QS triggers a cascade of transduction signals that results in a change of gene expression enabling the bacterial population to work in unison and behave collectively [62,63]. Besides the gene expression changes, QS allows the bacterial population to form biofilms or develop virulence factors to help bacteria avoid the immune response [64,65]. Combining gene expression changes and physiological aspects, such as biofilm synthesis, gives bacterial tolerance to antimicrobials [66].

Biofilm synthesis in *Burkholderia cenocepacia* is regulated by the QS signals *Burkholderia* diffusible signal factor (BDSF) and the N-acyl homoserine lactone (AHL) signal. It has been observed that the *cis*-14-methyl-2-pentadecenoic acid (**30**, Fig. 7) inhibits the production of BDSF and AHL signals by decreasing the expression of their synthase encoding genes. Therefore, this results in the inhibition of biofilm formation, virulence, and motility, but the growth rate is not affected [64]. Myristoleic acid (**7**) and palmitoleic acid (**8**) were also successfully tested against the *Acinetobacter baumannii* QS communication system. It was demonstrated that both FA successfully decreased the QS regulator abaR and consequently reduced the AHL signaling. As a result, **8** decreased biofilm formation up to 38% and **7** up to 24%. In addition, both **7** and **8** drastically reduced the motility of *A. baumannii* [67].

Another molecule that mediates the QS is the autoinducer-2 (AI-2) that is present in either Gram-negative or Gram-positive bacteria [68]. For example, Widmer et al. reported that **3**, **4**, **10**, and **24** decreased the AI-2 signaling in *Vibrio harveyi* resulting in inhibition of quorum sensing [69]. Lyngbyoic acid (**31**, Fig. 7) is another FA that also displays QS inhibitory activity. This FA, isolated from the cyanobacteria *Lyngbya majuscula*, affects the quorum sensing AHL signal of *P. aeruginosa* [33]. The protein LasR forms part of the AHL pathway, and it coordinates the expression of genes that encode for virulence factors [70]. Acid **31** strongly affects the AHL signal pathway through the AHL-binding domain of LasR [33].

The FAs mentioned above represent excellent examples of how FA exerts anti-QS activity in pathogenic bacteria. Agents that display anti-QS activity can eliminate QS signaling and prevent the formation of biofilms and virulence factors that confers pathogenicity to the bacteria. Therefore, discovering novel agents that inhibit QS is highly relevant to the field of drug development because they could be further tested as nonantibiotic therapies that can prevent infections and reduce the risk of bacteria acquiring resistance targeting the expression of pathogenic genes.

#### 4.2.3. Inhibition of efflux pumps

**Fig. 9.**
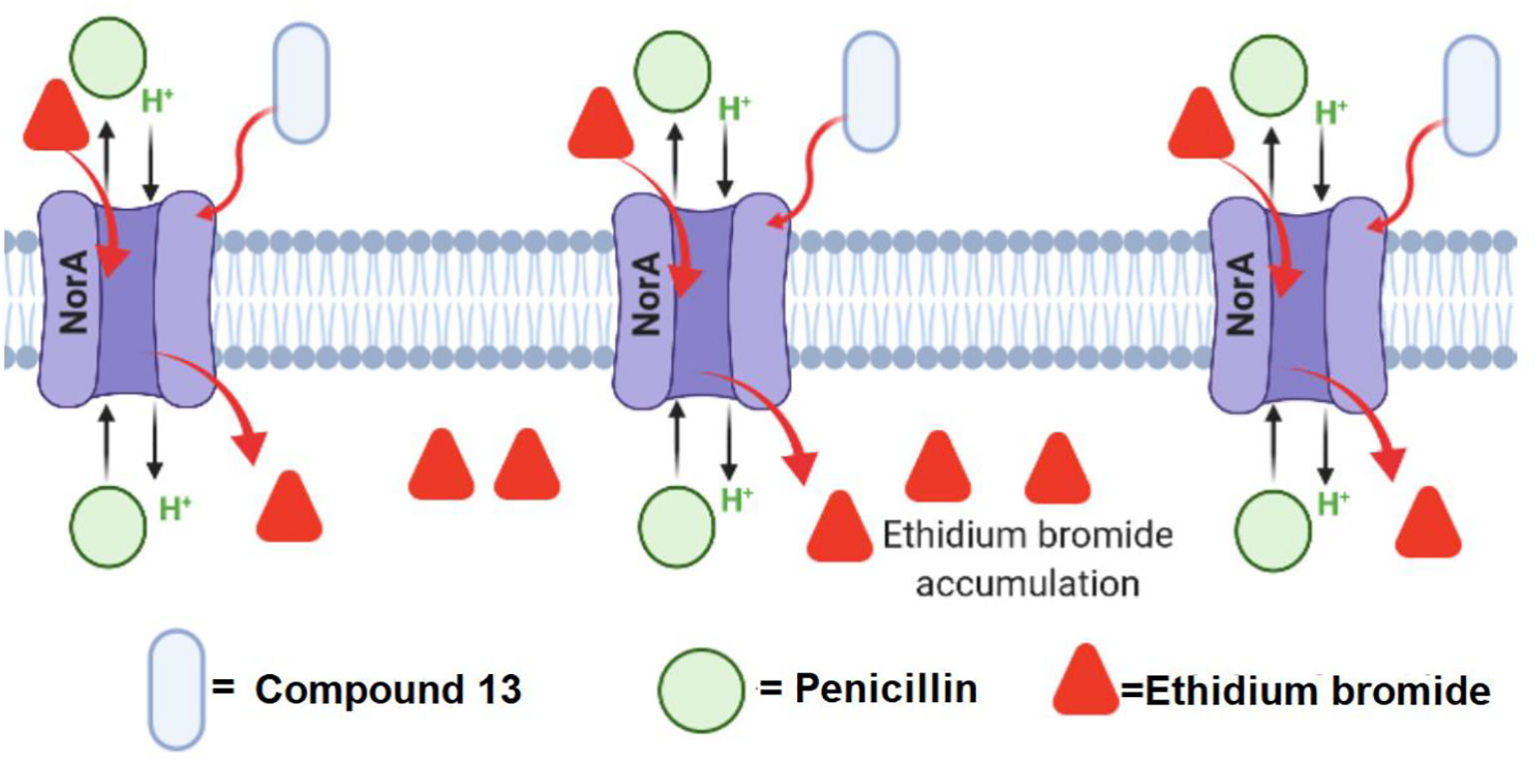
Schematic representation of the ethidium bromide permeabilization approach to measuring the inhibitory effect of 7,10-epoxyoctadeca-7,9-dienoic acid (**13**) combined with penicillin (light green) against the efflux pump NorA.This image shows the interaction between **13** (light blue oval) and the NorA efflux pump (surface protein in violet). It can be appreciated when **13** inhibits the efflux activity of NorA since ethidium bromide molecules (red triangles) accumulate inside the cytosol. The image was generated with BioRender.com. (For interpretation of the references to colour in this figure legend, the reader is referred to the web version of this article.)

Antibiotic efflux is one of several resistance mechanisms found in Gram-positive and Gram-negative bacteria [71,72]. This mechanism is mediated by surface proteins known as efflux pumps responsible for expelling out antibiotics with permeabilized bacterial cytoplasmatic membranes [73]. The furan fatty acid **13** displays inhibition of the NorA efflux pump in *S. aureus* [36]. This apparent inhibition could explain the improvement of penicillin’s antibacterial activity when combined with **13** (Fig. 9). The inhibition of efflux pumps by FA is definitely an exciting new field that needs to be further investigated.

## 5. Conclusions

This review has presented the enormous potential of using FA as the next-generation of antibacterial agents to treat bacterial infections in humans. Additionally, it has identified some chemical features needed to promote antibacterial activity against Gram-positive and Gram-negative bacteria, which could help develop novel antibacterial agents. Substantial progress has been made in understanding the relative potency and spectrum of antibacterial FA, particularly in identifying promising drug candidates. In recent years, biological research has been complemented, documenting possible traditional antibacterial FA mechanisms such as inhibition of DNA/RNA replication, inhibition of cell wall, protein synthesis inhibition, disruption of the cytoplasmic membrane, and inhibition of metabolic routes. Moreover, non-traditional mechanisms such as inhibition of HGT, QS, and efflux pumps have been documented as a possible mechanism of FA to reduce the bacterial resistance to antibiotics or decrease the bacterial development of virulence. We firmly believe that this review provides meaningful information that facilitates the understanding of mechanistic aspects that could explain the antibacterial activities of the most novel FA, knowledge that will help in the development of the next-generation of FA with efficacy as antibacterial agents.

## Figures and Tables

**Table 1.** Summary of relevant information regarding recent uFA and ring containing FA that have shown antibacterial activity against Gram-positive and Gram-negative bacteria.

FA	Chemical structure	Source	Type of formulation	Target bacteria	Inhibitory range	References
11	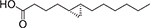	Isolated from *Labrenzia* sp. 011	Methanolic HCl	*E. coli, P. crassostreae*	Inhibition zones ranging from 2 to 5 mm	[34]
12	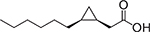	Isolated from *Labrenzia* sp. 011	Methanolic HCl	*E. coli, B. megaterium, P. crassostreae,* MRSA, *E. coli* I-11276b	Inhibition zones ranging from 2 to 10 mm	[34]
13	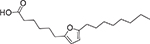	Synthetic	DMSO	*Bacillus brevis, Corynaebacterium glutamicum, S. mutans,* MRSA, Methicillin susceptible *S. aureus*	MIC ranging from 31.2 to 250 μg/mL	[35]

**Table 2.** Summary of unusual aFA that have shown antibacterial activity against Gram-positive and Gram-negative bacteria.

FA	Chemical structure	Source	Type of formulation	Target bacteria	Inhibitory range	References
14	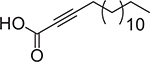	Synthetic	DMSO	*S. aureus, S. saprophyticus*, *B. cereus*, *K. pneumoniae, P. aeruginosa,* CIMRSA, CRSA	MIC ranging from 3.9 to 125 μg/mL	[9,37]
15	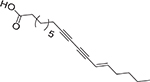	Isolated from *Thesium chinese*	Ethanol 95%, *v*/v	*P. gingivalis, F. nucleatum, S. mutans*	MIC ranging from 0.86 to 3.70 μg/mL	[38]
16	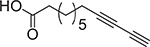	Isolated from *Thesium chinese*	Ethanol 95%, *v*/v	*P. gingivalis, F. nucleatun*	MIC ranging from 1.20 to 9.60 μg/mL	[38]
17	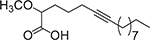	Synthetic	DMSO	*S. aureus,* CIMRSA, *E. coli*	MIC ranging from 62.5 to 500 μg/mL	[22]
18	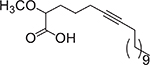	Synthetic	DMSO	*S. aureus,* CIMRSA, *E. coli*	MIC ranging from 31.3 to 1000 μg/mL	[22]
19	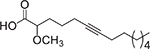	Synthetic	DMSO	*S. aureus,* CIMRSA	30–48 μg/mL	Unpublished results

## Abbreviations:

aFA: Acetylenic fatty acids
AHL: N-acyl homoserine lactone
AK: Amikacin
AMP: Ampicillin
BDSF: Burkholderia diffusible signal factor
CAZ: Ceftazidime
CIMRSA: Clinical isolates of methicillin-resistant *S. aureus*
CIP: Ciprofloxacin
CMC: Critical micelle concentration
CN: Cephalexin
CPM: Cefepime
CRSA: Ciprofloxacin-resistant *S. aureus*
CTX: Ceftriaxone
DHA: Docosahexaenoic acid
7,10-EODA: 7,10-Epoxyoctadeca-7,9-dienoic acid
EPA: Eicosapentaenoic acid
GEN: Gentamycin
HGT: Horizontal gene transfer
IPM: Imipenem
MBC: Minimum bactericidal concentration
MCFA: Medium-chain fatty acids
MDRSA: Multidrug-resistant *S. aureus*
MIC: Minimum inhibitory concentration
MRSA: Methicillin-resistant *Staphylococcus aureus*
NCL: Nanostructured lipid carrier
NIT: Nitrofurantoin
PG: Peptidoglycan
PIP: Piperacillin
PUFA: Polyunsaturated fatty acids
QS: Quorum sensing
SAR: Structure-activity relationship
SEM: Scanning electron microscopy
SFA: Saturated fatty acids
TEM: Transmission electron microscopy
TOB: Tobramycin
uFA: Unsaturated fatty acids
